# Bariatric surgery can acutely modulate ER-stress and inflammation on subcutaneous adipose tissue in non-diabetic patients with obesity

**DOI:** 10.1186/s13098-021-00623-w

**Published:** 2021-02-16

**Authors:** Rafael Ferraz-Bannitz, Caroline Rossi Welendorf, Priscila Oliveira Coelho, Wilson Salgado, Carla Barbosa Nonino, Rebeca A. Beraldo, Maria Cristina Foss-Freitas

**Affiliations:** 1 Division of Endocrinology and Metabolism, Department of Internal Medicine, Ribeirao Preto Medical School, University of Sao Paulo (USP), Avenida Bandeirantes, 3900-Vila Monte Alegre, Ribeirao Preto, SP 14049-900 Brazil; 2 Department of Surgery and Anatomy, Ribeirão Preto Medical School, University of São Paulo, Ribeirao Preto, SP Brazil; 3 Laboratory of Nutrigenomic Studies, Ribeirão Preto Medical School, University of Sao Paulo (USP), Ribeirao Preto, SP Brazil

**Keywords:** Obesity, Bariatric surgery, Roux-en-Y gastric bypass, Human adipose tissue, Endoplasmic reticulum stress, Oxidative stress, Inflammation

## Abstract

**Background:**

Bariatric surgery, especially Roux-en-Y gastric bypass (RYGB), is the most effective and durable treatment option for severe obesity. The mechanisms involving adipose tissue may be important to explain the effects of surgery.

**Methods:**

We aimed to identify the genetic signatures of adipose tissue in patients undergoing RYGB. We evaluated 13 obese, non-diabetic patients (mean age 37 years, 100% women, Body mass index (BMI) 42.2 kg/m^2^) one day before surgery, 3 and 6 months (M) after RYGB.

**Results:**

Analysis of gene expression in adipose tissue collected at surgery compared with samples collected at 3 M and 6 M Post-RYGB showed that interleukins [Interleukin 6, Tumor necrosis factor*-α* (*TNF*-α), and Monocyte chemoattractant protein-1(MCP1)] and endoplasmic reticulum stress (ERS) genes [Eukaryotic translation initiation factor 2 alpha kinase 3 (EIF2AK3) and Calreticulin (CALR)] decreased during the follow-up (P ≤ 0.01 for all). Otherwise, genes involved in energy homeostasis [Adiponectin and AMP-activated protein kinase (AMPK)], cellular response to oxidative stress [Sirtuin 1, Sirtuin 3, and Nuclear factor erythroid 2-related factor 2 (NRF2)], mitochondrial biogenesis [Peroxisome proliferator-activated receptor gamma coactivator 1-alpha (PGC1α)] and amino acids metabolism [General control nonderepressible 2 (GCN2)] increased from baseline to all other time points evaluated (P ≤ 0.01 for all). Also, expression of Peroxisome proliferator-activated receptor gamma (PPAR*ϒ*) (adipogenesis regulation) was significantly decreased after RYGB (P < 0.05). Additionally, we observed that PGC1α, SIRT1 and AMPK strongly correlated to BMI at 3 M (P ≤ 0.01 for all), as well as ADIPOQ and SIRT1 to BMI at 6 M (P ≤ 0.01 for all).

**Conclusions:**

Our findings demonstrate that weight loss is associated with amelioration of inflammation and ERS and increased protection against oxidative stress in adipose tissue. These observations are strongly correlated with a decrease in BMI and essential genes that control cellular energy homeostasis, suggesting an adaptive process on a gene expression level during the caloric restriction and weight loss period after RYGB.

*Trial registration* CAAE: 73,585,317.0.0000.5440

## Background

Obesity is recognized as one of the leading global health problems, and its management is a significant challenge for health services due to its relationship with metabolic complications, including Type 2 Diabetes (T2D), cardiovascular diseases and several types of cancer [1–4].

Currently, bariatric surgery is considered the best treatment for sustained weight loss and reduced comorbidities associated with obesity [5]. Especially Roux-en-Y gastric bypass (RYGB) is considered one of the most effective procedures, promoting both weight reduction and improvement of metabolism [5, 6]. The mechanisms underlying the remarkable effects induced by RYGB in decreasing body weight in controlling T2D and cardiovascular diseases remain uncertain. Some studies suggest that postoperative caloric restriction is sufficient to explain the metabolic effects of RYGB [7, 8].

The weight loss provided by RYGB directly affects the amount of body adipose tissue. Identifying adipose tissue as a metabolically active tissue in humans has positioned adipocyte as the target of many investigations. Several genes expressed mainly in adipose tissue are involved in various metabolic and endocrine functions, such as adipocyte development, lipid metabolism, glucose homeostasis and inflammatory responses [9–11]. The adipose tissue secretes several pro-inflammatory cytokines, such as interleukin-6 (IL6), tumor necrosis factor-a (TNF-a) and monocyte chemo-attracting protein (MCP-1), in addition, anti-inflammatory cytokines, such as adiponectin (ADIPOQ), which is associated with increased insulin sensitivity. [12]. Another specific adipose gene is the peroxisome proliferator-activated gamma receptor (PPAR*ϒ*) that plays a determining role in the distribution of body fat in humans [13, 14]. Another gene that is involved in the regulation of metabolism is PGC-1ɑ (PPARγco-activator-1). This one plays a critical role in maintaining glucose, lipid, and energy homeostasis and is likely involved in pathogenic conditions such as obesity and diabetes [15].

The inflammatory process, characteristic of obesity, is linked in many levels to the endoplasmic reticulum stress (ERS). When the ER becomes stressed due to the accumulation of newly synthesized unfolded proteins, the unfolded protein response (UPR) is activated [16]. Excessive UPR signaling is associated with obesity and metabolic dysfunction [17, 18]. One of the main monitors of the ER lumen is EIF2AK3 (Eukaryotic Translation Initiation Factor 2 Alpha Kinase 3), one of the first triggers of the ERS [19]. Moreover, other major effectors of the ERS response is ATF4 (activating transcription factor 4) [20]. ATF4 plays an important role in regulating obesity as well as glucose homeostasis in mammals [21].

In mammalian systems, Sirtuins such as SIRT1 (sirtunin 1) and SIRT3 (sirtunin 3) are NAD + protein-dependent deacylases, being indispensable energy sensors, and their function is intrinsically linked to cellular metabolism [22]. Several studies have reported that caloric restriction induces the expression of the SIRT1 [23, 24] and SIRT3 [25] and that obesity can reduce the expression of SIRT1 in humans [26, 27]. There are several other nutrient sensors besides sirtuins, such as AMP-activated protein kinase (AMPK), a nutrient and energy sensor that maintains energy homeostasis [28] and general control nonderepressible 2 (GCN2), a kinase that modulates response to amino acid starvation [29], both sensors activated by caloric restriction.

Studies have proposed that oxidative stress plays an important role in the metabolic syndrome's genesis [30, 31]. Also, obesity is associated with reduced expression of several antioxidant proteins [32]. The superoxide dismutases (SOD) represent the primary cellular defense against oxidative stress. Animal studies have shown that mice with increased Sod2 expression were protected from obesity-induced insulin resistance [33]. Furthermore, Nuclear factor erythroid 2 -related factor 2 (NRF2) is considered other fundamental factor in resistance to oxidative stress [34], and its relationship to obesity has been reported in several studies [35, 36], NRF2 being a promising target for the treatment of obesity [30]. Thus, the functional study of adipose tissue in humans is an excellent strategy to understand in more detail the effects of RYGB.

To assess whether genetic alterations post-bariatric surgery have a dynamism that can be linked to protective effects against the development of metabolic syndrome this study aimed to determine the effects of RYGB on adipose tissue in obese non-diabetic women after 3 and 6 months compared to baseline. This approach allowed us to determine that RYGB induces significant adipose tissue changes, which may be necessary for protection against metabolic complications.

## Materials and methods

### Subjects and study design

The cohort included 13 non-diabetic obese women who underwent gastric bypass surgery. The subjects were recruited using personal communication and social media attended at the Clinics Hospital of Ribeirao Preto -USP. Participants were selected from March 2018 to January 2019, and exclusion criteria included complications from the metabolic syndrome, malignancy, pregnancy and body weight over 140 kg. None of the participants in this study were using any medication that could interfere in the glycemic control. Three analyzes were carried out, one day before surgery and 3 and 6 months after the surgical procedure. The measurements included anthropometry, blood biochemistry and abdominal adipose tissue biopsy analysis.

All procedures in this study were performed following the ethical standards as laid down in the Declaration of Helsinki. Informed consent was signed and obtained from all subjects. The study protocol was approved by the Ethics Committee of the Ribeirao Preto Medical School at the University of Sao Paulo, Brazil (protocol number CAAE: 73585317.0.0000.5440).

#### Anthropometric assessment

Body weight (kg) was measured with an electronic Filizola scale of platform type with a maximum capacity of 300 kg and a precision of 0.1 kg. Fat mass and percent and fat-free mass were measured using direct segmental multi-frequency bioelectrical impedance analysis (BIA 310e Bioimpedance Analyzer-Biodynamics). Height was measured using a fixed stadiometer to the nearest 0.1 cm. Body mass index (BMI) was calculated as kg/m^2^ accordingly. Hip and waist circumferences were measured to the nearest 0.01 m using a non-stretchable measuring tape (3 M).

#### Biochemical analysis

Blood samples were obtained after 12-h fasting. The biochemical analyzes were performed in the central laboratory of the Clinics Hospital of Ribeirao Preto. Commercial kits were used to measure insulin (IMMULITE 2000), A1C (D-10 Hemoglobin Testing System, Bio-Rad), glucose, cholesterol, triglycerides, LDL and HDL (Wiener lab CMD 800ix2).

Insulin resistance was estimated from blood samples with the homeostasis model assessment for insulin resistance (HOMA-IR), calculated using the HOMA2 Calculator software (http://www.dtu.ox.ac.uk/homacalculator/) [37].

#### Biopsy, RNA extraction and qPCR

Biopsy of subcutaneous adipose tissue (80 mg) was taken after local anesthesia from 13 non-diabetic obese women after informed consent by a trained surgeon from Ribeirao Preto Medical School-USP. The first biopsy was systematically collected from the right side of the abdomen and the second biopsy from the left side of the abdomen. The adipose tissue was rapidly transported to the research laboratory. Tissue samples were rinsed in phosphate-buffered saline to remove adhering blood and after was snap-frozen in liquid nitrogen and stored at − 80 ºC until completion of the study.

Briefly, RNA was isolated from approximately 40 mg of tissue using Trizol reagent (Life Technologies^®^), following the 'manufacturer's instructions and confirmed to be free of proteins or phenol using UV spectrophotometry. The cDNA synthesis was conducted using the iScript cDNA Synthesis Kit (Bio-Rad^®^) using 1 μg of total RNA. The gene expression rate was then evaluated by quantitative real-time PCR (qPCR). Each reaction mixture containing 250 nM of each primer (sense and antisense), 25  ng of cDNA and SsoFast EvaGreen Supermix (Bio-Rad^®^) in a final volume of 10 μL was analyzed in a CFX96 Touch™ Real-Time PCR Detection System (Bio-Rad^®^) under the following amplification conditions: 50 °C-2 min, 95 °C-10 min, 40 cycles of 95 °C-15 s, 60 °C-20 s and 72 °C-30 s.

#### Statistics

The results are given as the mean ± S.D. (Standard deviation) or SEM (Standard error of the mean). Statistical analyses were performed using either paired t-test or one-way repeated measures ANOVA with posthoc Tukey as appropriate. Pearson correlation coefficients were calculated to quantify the relationships between BMI changes and mRNA expression of defined target genes. Values were considered to be statistically significant when the P ≤ 0.05. The statistical analysis was performed with SAS (SAS Institute), and graph construction was performed using Graphpad Prism (Graphpad Prism 8 for Mac).

#### Data and resource availability

The data sets generated during and/or analyzed during the current clinical trial are available from the corresponding author upon reasonable request.

## Results

### Participant characteristics

Fifteen participants were invited, and thirteen participants were included in the study. We evaluated 13 obese, non-diabetic patients (mean age 37.7 ± 8.2 years; 100% women; Height 1.64 ± 0.05 m; BMI 42.2 ± 4.2 kg/m^2^), that underwent Roux-en-Y gastric bypass (RYGB). The clinical and biochemical characteristics of the evaluated group are summarized in Table 1.

**Table 1:** Clinical characteristics of the study participants

Characteristic	Before surgery	After 3 months	After 6 months
	(n = 13)	(n = 13)	(n = 13)
BMI (kg/m^2^)	42.2 ± 4.2	35.9 ± 4.7^*^	33.0 ± 2.9^###^
Body weight (kg)	113.4 ± 14.7	94.2 ± 13.9^*^	90.1 ± 12.4^##^
Waist circumference (cm)	123.2 ± 12.0	107.8 ± 7.7	107.2 ± 13.7^#^
Fat mass (kg)	52.8 ± 9.3	37.8 ± 9.1^*^	34.4 ± 7.1^###^
Lean mass (kg)	61.0 ± 5.9	56.4 ± 5.2	55.7 ± 6.1
Fasting glucose (mg/dL)	97.5 ± 25.1	87.0 ± 19.2	82.5 ± 11.6
A1C (%)	5.4 ± 0.7	5.2 ± 0.5	5.1 ± 0.4
Cholesterol (mg/dL)	165 ± 20.5	155.8 ± 27.1	160.9 ± 24.1
HDL cholesterol (mg/dL)	39.0 ± 9.5	39.1 ± 10.6	42.0 ± 10.2
LDL cholesterol (mg/dL)	105.7 ± 20.3	105.3 ± 20.1	100.4 ± 16.5
Triglycerides (mg/dL)	121.5 ± 67.0	86.5 ± 36.2	91.9 ± 40.1
Insulin (mU/L)	17.1 ± 7.7	9.0 ± 5.0	7.5 ± 4.4^#^
HOMA-IR	2.0 ± 1.0	1.1 ± 0.6^*^	0.9 ± 0.5^#^
Systolic BP (mmHg)	136 ± 9.6	113 ± 5.4***	114 ± 7.5^###^
Diastolic BP (mmHg)	102 ± 6.8	76 ± 5.4***	78.5 ± 6.4^###^
% of weight loss	NA	− 17%	− 20.50%
% blood glucose reduction	NA	− 12.90%	− 17.50%
% Cholesterol reduction	NA	− 5.50%	− 2.50%
% Triglycerides reduction	NA	− 30.90%	− 26.60%
% LDL reduction	NA	− 0.40%	− 5.00%
% HDL change	NA	− 0.25%	7.40%

Values are given as mean ± SD. One-way ANOVA, post-test Tukey

*BMI* body mass index, *A1C* hemoglobin A1C, *HDL* high-density lipoprotein, *LDL* low-density lipoprotein, *HOMA-IR* homeostatic model assessment-insulin resistance; *NA* not applicable

^*^^,#,$^p < 0.05; **^,##,$$^p < 0.01; ***^,###,$$$^p < 0.001

^*^ Baseline vs. 3 months

^#^ Baseline vs. 6 months

^$^ 6 months vs. 3 months

### Effects of RYGB on body composition, biochemicals parameters and blood pressure

**Fig. 1:**
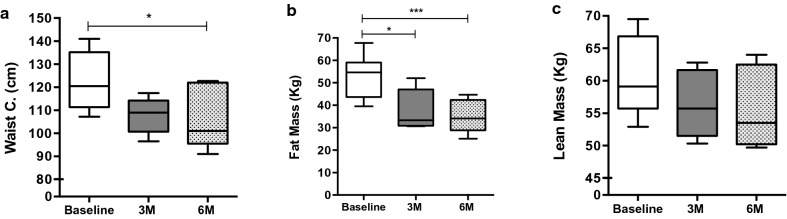
Anthropometric and body composition trajectories at baseline, 3 and 6 months after RYGB. **a** Waist circumference. **b** Fat mass. **c** Lean Mass. Significance determined by one-way ANOVA and Tukey post hoc test. Data are presented in box plot. N = 13 participants. *p < 0.05, ***p < 0.001

After RYGB, we observed a significant reduction in body weight and BMI after 3 months (3 M) (− 16.9%, P < 0.05; − 14.9%, P < 0.05 respectively) and 6 months (6 M) (− 20.5%, P < 0.01; − 21.8%, P < 0.001 respectively) Table 1. Waist circumference had not changed at 3 M, but after 6 M decreased by − 12.9% (P < 0.05). In addition, fat mass was decreased by -28.4% (P < 0.05) at 3 M and − 34.8% (P < 0.001) at 6 M. However, there was no difference in lean mass Fig. 1.

Despite having a downward trend fasting glucose levels and A1C, did not show significant differences after RYGB, Table 1. The total cholesterol, HDL cholesterol, LDL cholesterol and triglycerides did not show significant differences after RYGB, Table 1. Fasting insulin levels did not show significant change at 3 M; however, in 6 months, there was a significant reduction of 56.1% (P < 0.05), Table 1.

Interestingly, we observed a significant reduction in insulin resistance at 3 M (44.2%, P < 0.05) and at 6 M (53.8%, P < 0.05), as measured by the HOMA-IR index, Table 1. Blood pressure was reduced at 3 M (− 16.9% systolic BP; − 25.4% diastolic BP, P < 0.001 for all) and 6 M (− 16.1% systolic BP; − 23% diastolic BP, P < 0.001 for all), Table 1.

### Time course effects on expression of genes in subcutaneous adipose tissue

To identify possible changes in adipose tissue gene expression in non-diabetic obese individuals before and after RYGB, we performed a screening of several genes on the fat biopsies. We investigated interleukin genes, genes involved in energy homeostasis, adipogenesis, mitochondrial biogenesis, ERS and amino acid metabolism, summarized in Additional file 1: Table S1. Below we report the difference in the gene expression fold change between each time point evaluated.

**Fig. 2:**
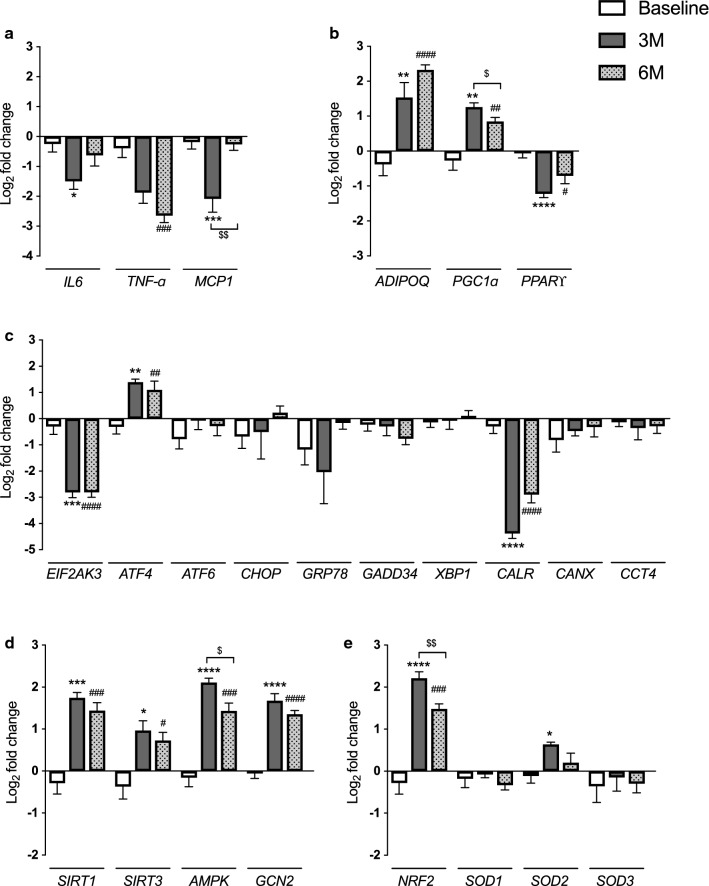
Gene expression signatures in the adipose tissue of obese individuals at baseline, 3 and 6 months after RYGB. The mRNA levels of genes of interleukins (**a**), genes associated with the metabolic function of adipose tissue (**b**), genes related to ER stress (**c**), genes associated with cellular energy sensors (**d**) and genes related to oxidative stress response (**e**). N = 13 samples per time. The fold changes were converted into log 2 values for comparison. Data are presented as means ± SEM. One-way ANOVA post-test Tukey. ****p < 0.0001, ***p < 0.001, **p < 0.01, and *p < 0.05

Three months (3 M) after RYGB was long enough to cause a decrease in the expression levels of *IL6* (Interleukin 6) (− 1.25 ± 0.4, P = 0.0175) and *MCP1* (monocyte chemoattractant protein 1) (− 1.90 ± 0.4, P = 0.0008) while a decrease in the expression of *TNF-ɑ* (Tumor necrosis factor ɑ) was observed only after 6 M (− 2.25 ± 0.4, P = 0.0001) in adipose tissue, Fig. 2a.

Additionally, we observed an increase in *ADIPOQ* (adiponectin) expression after 3 M (1.91 ± 0.5, P = 0.0051) and a more pronounced increase after 6 M (2.70 ± 0.4, P < 0.0001). Similarly, levels of *PGC1ɑ* (PPARγ coactivator-1) was increased after 3 M (1.53 ± 0.4, P < 0.0039) and 6 M (1.11 ± 0.3, P = 0.0096) of RYGB, Fig. 2b. In contrast, mRNA levels of *PPARϒ* (Peroxisome proliferator-activated receptor gamma) were reduced after 3 M (− 1.15 ± 0.2, P < 0.0001) and 6 M (− 0.64 ± 0.2, P = 0.0127), Fig. 2b.

Next, we examined the subcutaneous adipose tissue expression of genes involved in the ERS. *EIF2AK3* (Eukaryotic Translation Initiation Factor 2 Alpha Kinase 3) was decreased at 3 M (− 2.51 ± 0.5; P = 0.0001) and 6 M (− 2.50 ± 0.4; P < 0.0001). Also, the mRNA levels of *ATF4* (Activating Transcription Factor 4) was increased after 3 M (1.70 ± 0.4; P = 0.0017) and remained increased after 6 M (1.41 ± 0.4; P = 0.0051). Notably, we found that *CARL* (Calreticulin) expression was significant decreased at 3 M (− 4.08 ± 0.4; P < 0.0001) and 6 M (− 2.59 ± 0.4; P < 0.0001) after RYGB, Fig. 2c. The mRNA expression of other ERS-related genes did not show significant differences.

We also tested several genes related to the cellular response to oxidative stress and sensor of cellular energy homeostasis and amino acid concentration. In general, at 3 M after RYGB, increased expression of *SIRT1* (2.03 ± 0.4; P < 0.0002) and *SIRT3* (1.33 ± 0.5; P = 0.0177). At 6 M, the mRNA expression of *SIRT1* and *SIRT3* remained increased compared to the baseline (1.73 ± 0.3, P = 0.0002; 1.09 ± 0.4, P = 0.0205, respectively). Moreover, compared with baseline, the expression of *AMPK*, was higher at 3 M (2.27 ± 0.3; P < 0.001) and 6 M (1.58 ± 0.3; P = 0.0001) after RYGB. Finally, we verify that *GCN2* mRNA levels was increased after 3 M (1.73 ± 0.2; P < 0.001) and 6 M (1.41 ± 0.1; P < 0.001), Fig. 2d.

We sought to determine whether RYGB could induce changes in gene expression related to oxidative stress directly in adipose tissue. Notably, we found that *NRF2* expression was increased at 3 M (2.49 ± 0.4; P < 0.001) and 6 M (1.76 ± 0.3; P = 0.0002) compared to baseline. In addition, *SOD2* expression was increased in adipose tissue after 3 M (0.75 ± 0.2; P = 0.0161). However, we found no differences in expression at *SOD1* and *SOD3* in adipose tissue after RYGB, Fig. 2e. These data demonstrate the genetic dynamism of adipose tissue post-RYGB.

### BMI is positively correlated with PGC1ɑ, SIRT1, AMPK and Adiponectin expression in human adipose tissues

We performed correlation analysis between gene expression in adipose tissue samples and the biochemical and anthropometric variables to address possible associations before and after RYGB.

Interestingly, we noted that after 3 M, the expression of *PGC1ɑ, SIRT1,* and *AMPK* was positively correlated with the BMI changes in the same period (Pearson's correlation r = 0.961, P = 0.009; r = 0.958, P = 0.010; r = 0.947, P = 0.014 respectively), Table 2. Similar patterns of significant correlations were found between the gene expression of *ADIPOQ* and *SIRT1* after 6 M of RYGB and changes in BMI at 6 M (r = 0.950, P = 0.001; r = 0.844, P = 0.016 respectively), Table 2. These data collectively suggest that the relationship between the expression of genes related to the control of energy homeostasis and mitochondrial biogenesis may be dependent on post-RYGB time dynamics in adipose tissue.

**Table 2:** Correlations between BMI changes and delta of gene expression in adipose tissue at 3 and 6 months after RYGB

	Correlation statistics			Correlation statistics	
	Pearson’s r	p value		Pearson’s r	p value
3 months			6 months		
* IL6*	0.362	0.548	* IL6*	-0.305	0.5046
* TNFα*	0.344	0.570	* TNFα*	-0.555	0.1954
* MCP1*	0.497	0.394	* MCP1*	0.183	0.6942
* ADIPOQ*	-0.066	0.915	***ADIPOQ***	**0.950**	**0.0010**
***PGC1α***	**0.961**	**0.009**	* PGC1α*	0.386	0.3915
* PPARγ*	0.384	0.523	* PPARγ*	0.591	0.1623
* EIF2AK3*	0.511	0.379	* EIF2AK3*	0.165	0.7233
* ATF4*	0.648	0.237	*ATF4*	-0.373	0.4097
* ATF6*	0.410	0.492	*ATF6*	0.677	0.0945
* CHOP*	0.254	0.680	*CHOP*	-0.286	0.5332
*GRP78*	0.261	0.671	*GRP78*	0.46	0.2987
*GADD34*	-0.554	0.332	*GADD34*	-0.05	0.9141
*XBP1*	-0.609	0.275	*XBP1*	0.232	0.6164
*CALR*	0.004	0.994	*CALR*	0.416	0.3523
*CANX*	0.490	0.401	*CANX*	0.743	0.0554
*CCT4*	0.637	0.247	*CCT4*	0.272	0.5539
***SIRT1***	**0.958**	**0.010**	***SIRT1***	**0.844**	**0.0169**
*SIRT3*	0.407	0.496	*SIRT3*	0.489	0.2646
***AMPK***	**0.947**	**0.015**	*AMPK*	0.692	0.0846
*GCN2*	-0.511	0.379	*GCN2*	0.278	0.5459
*NRF2*	0.127	0.839	*NRF2*	-0.014	0.9757
*SOD1*	-0.342	0.573	*SOD1*	-0.527	0.2232
*SOD2*	-0.710	0.178	*SOD2*	-0.112	0.8102
*SOD3*	-0.466	0.428	*SOD3*	0.105	0.8227

IL6: Interleukin 6; TNF-ɑ: *Tumor necrosis factor*-α; MCP-1: Monocyte chemoattractant protein-1; PGC1ɑ: Peroxisome proliferator-activated receptor gamma coactivator 1-alpha; PPARϒ: Peroxisome proliferator activated receptor gamma; EIF2AK3: Eukaryotic translation initiation factor 2 alpha kinase 3; ATF4: Activating Transcription Factor 4; ATF6: Activating Transcription Factor 6; CHOP: C/EBP homologous protein; GRP78: Heat shock protein family A (Hsp70) member 5; GADD34: growth arrest and DNA damage-inducible protein; XBP1: X-box binding protein 1; CARL: Calreticulin; CANX: Calnexin; CCT4: Chaperonin Containing TCP1 Subunit 4; SIRT1: Sirtuin 1; SIRT3: Sirtuin 3; AMPK: AMP-activated protein kinase; GCN2: General control nonderepressible 2; NRF2: Nuclear factor erythroid 2-related factor 2; SOD1: Superoxide dismutase 1; SOD2: Superoxide dismutase 2, SOD3: Superoxide dismutase 3

## Discussion

This study investigated the impact of RYGB on the dynamic of gene expression in adipose tissue in non-diabetic obese humans. Our study provides interesting evidence of the acute effect of RYGB on human adipose tissue at the molecular level, we also, describe the associations of gene expression with metabolic improvement provided by the surgical procedure.

As described in the literature, our study confirms the pronounced effect of RYGB on BMI and fat mass reduction after 3–6 months [38, 39]. As expected, this was accompanied by metabolic improvements such as decreased fasting insulin levels, HOMA-IR and blood pressure. Our findings agree with some authors that described a rapid improvement in insulin resistance, mainly reducing insulin levels after RYGB [40, 41]. Our participants also had a 65% improvement in HOMA-IR after 6 months, very similar to the results reported by Rao and colleagues after the same period [42]. Previous studies have also demonstrated that bariatric surgery markedly reduces the risk of cardiovascular disease, including the decrease in hypertension associated with obesity [38, 43, 44]. We confirm earlier findings, showing that in non-diabetic and mildly hypertensive obese women, systolic and diastolic blood pressure decreased significantly after 3 and 6 months of RYGB. These observations are of interest because they demonstrate the same effects of metabolic improvement, characteristic of RYGB, in individuals with class III obesity and hypertension but no diabetes mellitus.

We also performed an extensive analysis of gene expression signatures in the adipose tissue of obese individuals before and 3 and 6 months after RYGB. Molecular analysis revealed that RYGB decreased the expression of genes involved in the inflammatory process, mainly *IL6* and *MCP1*, after 3 M and TNF-ɑ after 6 M, concomitant to an increased expression *ADIPOQ* at both times, an anti-inflammatory adipokine. Obesity is a pro-inflammatory condition in which adipocytes and immune cells residing in adipose tissue contribute to the increase in circulating levels of pro-inflammatory cytokines [45]. Inflammatory markers such as *IL6, MCP1* and *TNF-ɑ* are secreted by adipocytes and are directly associated with high BMI, obesity and insulin resistance [46–48]. *ADIPOQ* was positively correlated with BMI [49] and also has an important insulin-sensitizing effect [50]. Our findings showed that there are genetic changes of *IL6*, *MCP1* and *TNF-ɑ* in adipose tissue in the acute phase after RYGB, a result that was not observed in a study that evaluated the gene expression of these interleukins after 1 year [51]. However, our findings agree with a previous study that reported increased adiponectin expression after 6 M [52]. Given that inflammatory pathways can exert opposing or redundant functions, the decreased expression of genes encoding adipokines at adipose tissue, may be responsible for the improved metabolic and decreased insulin resistance seen at RYGB.

In our study, specific adipose genes such as *PPARϒ* and *PGC-1ɑ* had a significant alteration in gene expression after 3 and 6 M. Changes in adipose tissue metabolism's main regulators have significant implications for energy metabolism and the response to insulin sensitivity. The reduction in *PPARϒ* expression observed in our study is in harmony with other authors who showed a decrease in *PPARϒ* expression after bariatric surgery [53, 54]. The negative regulation of *PPARϒ* in adipose tissue after RYGB suggests adipogenesis inhibition and an improvement in insulin sensitivity [55]. Other adipogenesis related genes such as *PGC-1ɑ* was significantly upregulated post-RYGB. Similar changes have been reported during weight loss after RYGB, which was associated with the improvement of insulin sensitivity [56]. Our findings suggest that acutely after RYGB, adipose tissue has a decrease in PPAR*ϒ* adipose activity, while PGC-1ɑ shows upregulation and these changes was observed together with an improvement in insulin sensitivity.

Activation of ERS is known to exercise profound effects on various metabolic processes. We found that markers of ERS such as *EIF2AK3* and *CALR* significantly decreased gene expression after RYGB. Like showed by Mosinski et al., in animal model, *PERK* (*EIF2AK3*) gene expression was reduced after RYGB [57]. Gregor et al., reported a decrease in ERS in human samples of adipose tissue and liver [58]. However, they did not specifically study the gene expression of *EIF2AK3*. In contrast to Gregor et al. 's findings, we did not see any change in the *GRP78* gene expression after 3 M and 6 M in our study.

Interestingly, we found that *CARL* expression was decreased after RYGB. Calreticulin is found in several parts of the cell, including the ER. In addition, calreticulin plays a role in ensuring the proper folding of newly formed proteins in the ER [59]. We believe that we are the first group to describe the effects of RYGB under the expression of calreticulin in adipose tissue because no data are found in the literature to draw a parallel to our results. Together, these data demonstrate a significant regulation of ER stress in weight loss provided by RYGB and a possible link with the reduction of inflammation and metabolic improvement [16].

Sirtuins regulate the aging process and are present in critical tissues such as adipose tissue to mediate physiological adaptability to diets [22]. Here we showed that expression of *SIRT1* and *SIRT3* were enhanced in adipose tissue post-RYGB. In line with our observations, Moschen et al., demonstrated that *SIRT1* and *SIRT3* mRNA expression was higher in subcutaneous adipose tissue 6 months after laparoscopic adjustable gastric banding surgery. In addition, Pedersen et al. reported increased expression of *SIRT1* in adipose tissue biopsies from human volunteers submitted to 6 days of total fasting [26]. Our results supported by data in the available literature suggest that Sirtuins may play an important role in the beneficial effects of calorie restriction provided by the acute phase of RYGB.

AMPK is a nutrient and energy sensor that maintains energy homeostasis [60]. We have shown here that *AMPK* expression increased after 3 M and 6 M of RYGB. Our results are consistent with previous studies showing an association between improved AMPK activity and insulin sensitivity in individuals after RYGB weight-loss surgery [61]. *GCN2* was first discovered as a critical sensor of amino acid depletion [62]. In amino acid deprivation, GCN2 phosphorylates eIF2α, leading to the inhibition of general protein synthesis while increasing the translation of specific transcription factors, such as ATF4 [63]. Consistently we found that *GCN2* mRNA expression was markedly increased after 3 M and 6 M of RYGB and an increased expression of *ATF4*. This outcome suggests that the acute effects induced by post-RYGB calorie restriction consistently act to increase the gene expression of classic nutritional sensors like AMPK but also GCN2, a specific amino acid sensor.

We also examined the activity of oxidative stress markers at adipose tissue due to its association in the genesis of the metabolic syndrome [30] and obesity [31]. Here we showed an upregulation of *NRF2* in adipose tissue after RYGB. NRF2 plays a critical role in adipose tissue working as a primary cellular defender against oxidative stress's cytotoxic effects [64]. Our findings agree with studies using animal model, which showed *NRF2* expression was significantly increased after RYGB [65]. Furthermore, *SOD2* expression, another important mediator against oxidative stress, is increased in adipose tissue after 3 M of RYGB. Some studies indicate that caloric restriction reduces oxidative stress by activation of SOD2 [66, 67], but we have found no studies that describe a SOD2 response to the effects of RYGB. Our results revealed rapid changes in adipose tissue gene expression within 6 M after RYGB. The current data set suggests that the acute phase of RYGB promotes a substantial metabolic change in human adipose tissue creating a potentially beneficial physiological status associated with weight loss and insulin sensitivity.

Finally, we noted a strong correlation between BMI alteration and expression of *PGC1ɑ*, *SIRT1*, *AMPK* after 3 M and *ADIPOQ* and *SIRT1* after 6 M of RYGB. The data presented here extend the findings of the effects of RYGB on adipose tissue, providing associative evidence between decreased BMI and genes that control cellular energy homeostasis, nutritional sensors, anti-inflammatory functions and insulin sensitivity, suggesting an adaptive process in the level of gene expression as a result of caloric restriction and weight loss after the RYGB.

There are some limitations to the current study that should be mentioned. We investigated only female patients, and we had a small sample size due to difficulties in accepting and performing biopsies of adipose tissue in our patients. Also, we only had access to subcutaneous fat removed from the abdominal region. With that, we concentrated our analyzes on this biological material. Due to the low amount of material collected, we were only able to analyze gene expression of the adipose tissue's gene expression, not confirm our findings at the protein level. This study did not analyze or include the potential influences of the incretin system on outcomes.

## Conclusion

Our study shows that RYGB has short-term effects on the dynamism of gene expression in adipose tissue in non-diabetic obese women and that these changes have the potential to modulate metabolic regulation, mainly the attenuated inflammatory response, decreased ERS, activation of genes that respond to oxidative stress and genes that inhibit adipogenesis and improve insulin sensitivity. Our discoveries of the molecular signatures for improving metabolic function after RYGB, especially under adipose tissue, may help develop new strategies to produce the same benefits as RYGB in patients not eligible for surgery.

## Supplementary information

**Additional file 1: Table S1.** Gene Expression of adipose tissue 3 and 6 months post RYGB compared with Baseline.

## Data Availability

The datasets used and/or analyzed during the current study are available from the corresponding author on reasonable request.

## Abbreviations

T2D: Type 2 Diabetes
RYGB: Roux-en-Y gastric bypass
IL6: Interleukin-6
TNF-a: Tumor Necrosis Factor-a
MCP-1: Monocyte Chemo-Attracting Protein 1
ADIPOQ: Adiponectin
PPAR*ϒ*: Peroxisome Proliferator-Activated Gamma Receptor
PGC-1ɑ: PPARγco-activator-1
ERS: Endoplasmic Reticulum Stress
UPR: Unfolded Protein Response
EIF2AK3: Eukaryotic Translation Initiation Factor 2 Alpha Kinase 3
CHOP: C/EBP homologous protein
GRP78: Heat shock protein family A (Hsp70) member 5
GADD34: Growth arrest and DNA damage-inducible protein
XBP1: X-box binding protein 1
CARL: Calreticulin
CANX: Calnexin
CCT4: Chaperonin Containing TCP1 Subunit 4
ATF4: Activating Transcription Factor 4
SIRT1: Sirtunin 1
SIRT3: Sirtunin 3
AMPK: AMP-Activated Protein Kinase
GCN2: General Control Nonderepressible 2
SOD1: Superoxide dismutase 1
SOD2: Superoxide dismutase 2, SOD3: Superoxide dismutase 3
NRF2: Nuclear Factor Erythroid 2 -Related Factor 2
HOMA-IR: Homeostasis Model Assessment for Insulin Resistance
