# Supplementary material for: Bariatric surgery can acutely modulate ER-stress and inflammation on subcutaneous adipose tissue in non-diabetic patients with obesity
Source: Diabetol Metab Syndr. 2021 Feb 16;13:19. doi: 10.1186/s13098-021-00623-w (PMC7887793; doi:10.1186/s13098-021-00623-w)
Supplement: Supplementary file 1 — Additional file 1: Table S1. Gene Expression of adipose tissue 3 and 6 months post RYGB compared with Baseline. [file 13098_2021_623_MOESM1_ESM.docx]

| **Table S1. Gene Expression of adipose tissue 3 and 6 months post RYGB compared with Baseline** | | | | | | |
| --- | --- | --- | --- | --- | --- | --- |
|  | **Baseline vs 3 months** | | **Baseline vs 6 months** | | **6 months vs 3 months** | |
| **Gene Name** | **Log_2_ Fold Change ± SEM** | **P Value** | **Log_2_ Fold Change ± SEM** | **P Value** | **Log_2_ Fold Change ± SEM** | **P Value** |
| *IL6* | -1.25 ± 0.47 | **0.0175** | -0.37 ± 0.45 | 0.4166 | -0.87 ± 0.48 | 0.1002 |
| *TNFα* | -1.48 ± 0.55 | 0.0566 | -2.25 ± 0.46 | **0.0001** | 0.77 ± 0.40 | 0.0866 |
| *MCP1* | -1.90 ± 0.46 | **0.0008** | -0.07 ± 0.35 | 0.8309 | -1.82 ± 0.43 | **0.0018** |
| *ADIPOQ* | 1.91 ± 0.59 | **0.0051** | 2.70 ± 0.46 | **< 0.0001** | -0.79 ± 0.39 | 0.0700 |
| *PGC1α* | 1.53 ± 0.45 | **0.0039** | 1.11 ± 0.38 | **0.0096** | 0.41 ± 0.17 | **0.0367** |
| *PPARγ* | -1.15 ± 0.21 | **< 0.0001** | -0.64 ± 0.23 | **0.0127** | -0.51 ± 0.27 | 0.0970 |
| *EIF2AK3* | -2.51 ± 0.50 | **0.0001** | -2.50 ± 0.43 | **< 0.0001** | -0.01 ± 0.28 | 0.9686 |
| *ATF4* | 1.70 ± 0.45 | **0.0017** | 1.41 ± 0.44 | **0.0051** | 0.29 ± 0.40 | 0.4852 |
| *ATF6* | 0.72 ± 0.64 | 0.2789 | 0.50 ± 0.57 | 0.3929 | 0.21 ± 0.54 | 0.6938 |
| *CHOP* | 0.17 ± 0.94 | 0.8572 | 0.91 ± 0.64 | 0.1686 | -0.74 ± 0.89 | 0.4259 |
| *GRP78* | -0.86 ± 1.19 | 0.4798 | 1.01 ± 0.82 | 0.2318 | -1.87 ± 1.03 | 0.0989 |
| *GADD34* | -0.06 ± 0.44 | 0.8906 | -0.53 ± 0.37 | 0.1690 | 0.46 ± 0.40 | 0.2737 |
| *XBP1* | 0.07 ± 0.36 | 0.8466 | 0.24 ± 0.30 | 0.4232 | -0.17 ± 0.37 | 0.6497 |
| *CALR* | -4.08 ± 0.46 | **< 0.0001** | -2.59 ± 0.43 | **< 0.0001** | -1.48 ± 0.41 | **0.0048** |
| *CANX* | 0.34 ± 0.75 | 0.6525 | 0.49 ± 0.67 | 0.4718 | -0.15 ± 0.46 | 0.7489 |
| *CCT4* | -0.21 ± 0.39 | 0.5825 | -0.15 ± 0.30 | 0.6238 | -0.06 ± 0.49 | 0.8984 |
| *SIRT1* | 2.03 ± 0.42 | **0.0002** | 1.73 ± 0.37 | **0.0002** | 0.30 ± 0.24 | 0.2517 |
| *SIRT3* | 1.33 ± 050 | **0.0177** | 1.09 ± 0.43 | **0.0205** | 0.23 ± 0.30 | 0.4447 |
| *AMPK* | 2.27 ± 0.35 | **< 0.0001** | 1.58 ± 0.32 | **0.0001** | 0.68 ± 0.24 | **0.0181** |
| *GCN2* | 1.73 ± 0.21 | **< 0.0001** | 1.41 ± 0.17 | **< 0.0001** | 0.39 ± 0.17 | 0.0990 |
| *NRF2* | 2.49 ± 0.44 | **< 0.0001** | 1.76 ± 0.37 | **0.0002** | 0.73 ± 0.19 | **0.0033** |
| *SOD1* | 0.10 ± 0.34 | 0.7775 | -0.14 ± 0.30 | 0.6269 | 0.25 ± 0.15 | 0.1400 |
| *SOD2* | 0.75 ± 0.28 | **0.0161** | 0.31 ± 0.28 | 0.2791 | 0.43 ± 0.27 | 0.1470 |
| *SOD3* | 0.20 ± 0.66 | 0.7568 | 0.05 ± 0.55 | 0.9158 | 0.14 ± 0.36 | 0.6955 |

**Supplementary Table 1. Gene Expression of adipose tissue 3 and 6 months post RYGB compared with Baseline.** The table display the Log_2_ fold changes at baseline vs 3 months after RYGB, baseline vs 6 months after RYGB and 6 months vs 3 months. Significant P Values are shown in bold. All values are given as delta Log_2_ fold change ± SEM. Unpaired Student’s t test was performed. N=13 individuals.

Abbreviations: IL6: Interleukin 6; TNF-ɑ: Tumor necrosis factor-α; MCP-1: Monocyte chemoattractant protein-1; PGC1ɑ: Peroxisome proliferator-activated receptor gamma coactivator 1-alpha; PPARϒ: Peroxisome proliferator activated receptor gamma; EIF2AK3: Eukaryotic translation initiation factor 2 alpha kinase 3; ATF4: Activating Transcription Factor 4; ATF6: Activating Transcription Factor 6; CHOP: C/EBP homologous protein; GRP78: Heat shock protein family A (Hsp70) member 5; GADD34: growth arrest and DNA damage-inducible protein; XBP1: X-box binding protein 1; CARL: Calreticulin; CANX: Calnexin; CCT4: Chaperonin Containing TCP1 Subunit 4; SIRT1: Sirtuin 1; SIRT3: Sirtuin 3; AMPK: AMP-activated protein kinase; GCN2: General control nonderepressible 2; NRF2: Nuclear factor erythroid 2-related factor 2; SOD1: Superoxide dismutase 1; SOD2: Superoxide dismutase 2, SOD3: Superoxide dismutase 3.
